# Fragmentation-Induced Disassembly and Reaggregation of α‑Synuclein Amyloid Fibrils

**DOI:** 10.1021/acschemneuro.5c00895

**Published:** 2026-05-20

**Authors:** Fritjof Havemeister, Vesa Halipi, Marziyeh Ghaeidamini, Elin K. Esbjörner

**Affiliations:** Division of Chemical Biology, Department of Life Sciences, 11248 Chalmers University of Technology, Kemivägen 10, Gothenburg 412 96, Sweden

**Keywords:** α-synuclein, amyloid kinetics, fragmentation, fibril morphology, fibril stability, polymorphism, thioflavin-T

## Abstract

Aggregation of the protein α-synuclein (α-syn) is a defining pathological characteristic of Parkinson’s disease (PD). Kinetic studies have provided increasingly detailed insights into the mechanisms of α-syn aggregation, highlighting the contributions of secondary nucleation and elongation to fibril growth. However, the understanding of the role of fibril breakage (fragmentation) remains sparse. We therefore established a modified thioflavin-T (ThT) kinetic assay in which ultrasonication steps were introduced when the conversion of α-syn monomers into amyloid fibrils had reached the plateau. This triggered, expectedly, fibril fragmentation but also rapid partial dissociation of the α-syn fibrils and subsequent elongation-dominated fibril regrowth, the kinetics of which could be monitored by ThT and were found to proceed until steady state was reestablished. Interestingly, the regrowth of α-syn variants A30P, E46K, and A53T, but not wild type or variant H50Q, resulted in significant increases in ThT fluorescence even though the residual monomer concentration at steady state was unaffected and no new monomers were added to the assayed systems. Furthermore, for these variants, which are all associated with early-onset PD, the residual monomer concentration was consistently higher than for wild-type α-syn and the late-onset variant H50Q, suggesting differences in monomer-fibril equilibria. Altogether, our study shows that α-syn amyloid fibrils are capable of undergoing structural evolution of a type that alters their ThT binding, highlights the role of fragmentation in expediating such maturation processes, and points out a putative connection between propensity of structural conversion, decreased fibril stability, and early onset of Parkinson’s disease.

## Graphical abstract

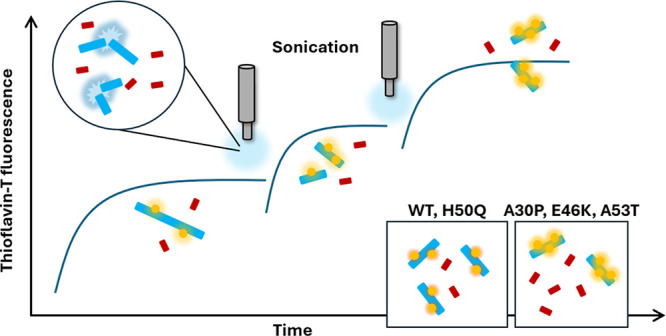

## Introduction

α-synuclein (α-syn) is a 14 kDa intrinsically disordered protein encoded by the human SNCA gene. Under physiological conditions, α-syn is mostly localized to nerve terminals^1^ where it is thought to have a role in the regulation of synaptic vesicle organization and neurotransmitter release,^2^ whereas under pathological conditions, the protein becomes prone to aggregation, which ultimately leads to the formation of cytosolic amyloid fibril inclusions in afflicted neuronal types. These inclusions are characteristic hallmarks of neurodegenerative disorders such as Parkinson’s disease,^3^ multiple system atrophy,^4^ and Lewy body dementia.^5^ Understanding the pathways and mechanisms of α-syn’s misfolding and aggregation is important to better describe, at a molecular and chemical level, the disease process and thereby aid towards the much-needed development of disease-modifying treatments.

Amyloid fibrils are self-assembled linear protein polymers with a common cross-β architecture.^6^ They form via reaction steps that involve primary and secondary nucleation, elongation, and fragmentation—processes that are nowadays well-described by kinetic models.^7,8^ However, amyloid formation is also a highly polymorphic process in that one protein can adopt many fibrillar and oligomeric states,^9^ which can coexist within samples^10,11^ and whose distributions and abundances are highly dependent on factors such as variations in reaction conditions^12,13^ or sequence mutations.^14,15^ Recent advances in structural biology have provided significant insights into the molecular architecture of various fibril polymorphs,^16^ including disease-specific variants isolated from postmortem brain samples,^17,18^ which thus represent the end points of long disease processes. However, understanding why certain environments and reaction conditions favor the formation of certain polymorphs remains a challenge. Furthermore, recent observations have shown that amyloid polymorphs and their relative distributions within a sample can evolve quite drastically over time and that such processes can include transiently populated structures.^10,19^ This manuscript focuses on the role of fragmentation in α-syn aggregation and explores how fragmentation processes can contribute to shape the mature fibril structure.

α-syn is a 140-amino-acid-long protein featuring an amphipathic and lipid-binding N-terminus and a C-terminal acidic tail.^20^ To date, there are at least 134 high-resolution structures of α-syn fibrils formed under different *in vitro* and *in vivo* conditions,^14^ emphasizing that the protein exhibits a highly polymorphic aggregation behavior. Most of these structures report an intricately folded amyloid core spanning, approximately, residues 36–100, as indicated in Figure 1A. Some structures indicate further engagement of an N-terminal segment (ca. residues 10–25), while the C-terminal tail consistently remains disordered. Studies of families with a history of Parkinson’s disease have identified a series of mutations that cause hereditary Parkinson’s disease with both early (A30P, E46K, and A53T) and late (H50Q) onsets.^5,21–23^ Notably, all of these mutations except A30P are located in the β-sheet core of α-syn fibrils or at the interface between protofilaments^24,25^ and they may hence influence not only the rates and mechanisms of α-syn assembly^26,27^ but also fibril structure^21,28,29^ and propensity to adopt polymorphic states.

The aggregation of α-syn *in vitro* is intrinsically extremely slow and typically requires surfaces or interfaces,^30^ agitation,^31^ lowering of pH or screening of electrostatic repulsion,^32,33^ or the addition of preformed seeds^34^ to overcome the primary nucleation barrier, which has, additionally, been suggested to involve the formation of a rather large critical nucleus.^35^ Studies in which this barrier has been overcome by different means have pointed out the importance of secondary nucleation as a major source of new α-syn aggregates.^33,36^

Amyloid formation does, to some extent, proceed via templated aggregation (e.g., the addition of monomers to a growing fibril end preserves its original structure).^37^ It is, however, increasingly realized that templating can be strongly dictated by solution conditions, rather than being entirely guided by the actual structure of the seed, especially during secondary nucleation,^38,39^ which is a major source of new aggregates in these reactions.^33,40^ Not even fibril elongation may always lead to faithful structural replication of a given fibril strain.^41^ The role of fragmentation, or breakage, of fibrils in shaping polymorph composition remains largely unexplored, despite its potential to influence fibril growth^42^ and disassembly,^43,44^ which is an underlying prerequisite for structural conversion of existing fibrils.

In this work, we have studied the aggregation kinetics and fibril conversion efficiencies in WT and mutant (A30P, E46K, H50Q, and A53T) α-syn samples. Furthermore, we introduced an ultrasonication step to fragment fibrils when the kinetic reactions approached steady state and observed how this resulted in mutant-specific patterns of disassembly and reaggregation. We found significant differences among the variants with respect to their residual monomer fractions and, hence, fibril conversion, but also in their reaggregation behavior following fragmentation. We rationalize our findings in relation to the disease association of the variants and suggest that early-onset Parkinson’s disease-associated α-syn mutations such as A30P, E46K, and A53T have a higher propensity of polymorph conversion than the wild-type protein.

## Material and Methods

### Expression and Purification of WT and Mutant α-syn

Recombinant WT α-syn was expressed in BL21­(DE3)­pLysS *Escherichia coli*, recombinant A30P, H50Q, A53T α-syn in BL21­(DE3), and recombinant E46K α-syn in BL21­(DE3)­pLysE *E. coli*. After harvesting the *E. coli*, the proteins were purified by acid precipitation,^45^ followed by ion exchange (Cytiva HiTrap Q 5 mL Fast Flow) and size exclusion (Cytiva HiLoad 16/600 Superdex 75 pg) chromatography using a Bio-Rad NGC Quest 10 Plus chromatography system. Purified WT and mutant α-syn were aliquoted and stored in monomeric form at −80 °C until further use.

### Formation of Preformed Fibrils (PFFs)

Preformed fibrils (PFFs) of WT and mutant α-syn were prepared and stored as described by Polinski et al.^46^ (e.g., the Michael J. Fox foundation protocol) with the difference of using Tris buffer instead of DPBS. Briefly, 347 μM solutions of WT or mutant α-syn in 100 mM NaCl, 0.1% NaN_3_, 20 mM Tris–HCl pH 7.4 were incubated for 7 days at 37 °C in an Eppendorf Thermomixer C set at 1000 rpm. This resulted in amyloid formation as verified by circular dichroism (CD), atomic force microscopy (AFM), and in a reduction in residual monomer as measured by absorption spectroscopy and confirmed by SDS-PAGE. The PFFs were aliquoted, frozen on dry ice, and stored at −80 °C until used in seeded aggregation kinetics experiments.

### Aggregation Kinetics Assays

Immediately prior to each experiment, aliquots of WT and mutant α-syn were thawed and purified by size exclusion chromatography (Superdex 75 10/300 GL) to remove potential aggregates and ensure monomeric starting solutions (Figure S1). Solutions containing 50 μM of monomeric WT or mutant α-syn, 2.5 μM of the corresponding PFF type, and 20 μM of thioflavin-T (ThT) were prepared in a 100 mM NaCl, 0.1% NaN_3_, and 20 mM Tris–HCl pH 7.4 buffer and distributed into the wells of Corning 96-well microplates (#3881), with 10 replicates per sample and 4 additional replicates that served as control sample (denoted as the continuous samples in the result section). The microplates were placed at 37 °C under quiescent conditions, in a FLUOstar Omega microplate reader, and ThT fluorescence was monitored until the plateau phase was reached. All the replicate samples were subsequently withdrawn from the plate and pooled into one sample per variant. Small volumes were set aside for the analyses described below. The control samples were always left in the well plate. The pooled samples were thereafter subjected to probe sonication to fragment fibrils (20% amplitude, 5 s on, 5 s off, for 20 s) using a Sonics VCX 750 ultrasonic liquid processor with a 2 mm tapered microtip (630-0417). Following sonication, additional volume was withdrawn for AFM analysis, and the remaining sample volume was rapidly (within 45 min) transferred back to the microplate, dispensing the same volume per well as initially distributed (hence the number of replicates decreased over the time course of the experiment). The microplate was returned to the plate reader, and the ThT fluorescence was monitored until a new plateau phase was reached. This aggregation-fragmentation cycle was repeated for a total of four rounds of aggregation (denoted A1–A4) and three rounds of sonication (denoted S1–S3), as schematically depicted in Figure 2A. In total, the samples were monitored over 383 h, with a sampling rate of six data points per hour and exposure time of 20 μs per data point, equaling a total excitation time of 0.046 s per sample. The number of replicates decreased by two to three wells per round due to the sample acquisition, liquid handling, and minor evaporation during the 400 h total time course of the experiment. ThT curves showing growth were fitted to single exponential functions (SI text) and AFM data, and absorbance data, the elongation rate constants, and fibril growth per minute (nm/min) were calculated. A more detailed description of the calculations can be found in the Supporting Information (Figure S18). Differences in end-point ThT intensities were evaluated for statistical significance using one-way ANOVA (*p* < 0.05).

### Residual Monomer Concentration

Samples from each aggregation plateau (end point of A1–A4) were centrifuged (30 min, 13,400 rpm) in an Eppendorf 5430R centrifuge to pellet aggregated material (the fibrils).^47,48^ The soluble material remaining in the supernatants was analyzed by SDS-PAGE (Invitrogen NuPAGE 4–12% Bis–Tris, 1 mm, mini protein gel) and absorbance measurements (ε_280_ = 5960 M^–1^ cm^–1^, Cary 4000 UV–Vis spectrophotometer) to estimate residual monomer content. ThT emission spectra were recorded to detect possible remaining amyloid aggregates in the supernatant, using a Cary Eclipse fluorimeter with 440 nm excitation and emission collected between 450 and 700 nm. Differences in fibril yield between variants were tested for statistical significance using one-way ANOVA (*p* < 0.05), and relations between fibril yield and end-point ThT intensities were analyzed using Pearson′s correlation test.

### Atomic Force Microscopy

Pelleted fibrils (see above) were resuspended in MQ water, and thereafter centrifuged again, and then subjected to a brief acid wash (6 mM HCl) to disentangle fibrils and detach any residual monomers stuck to the fibril surface, and a final centrifugation step to remove the acid as described by Wilkinson et al.^49^ The resuspended fibril sample was deposited onto freshly cleaved mica and allowed to settle for 4 min. The mica was thereafter rinsed 10× with MQ water and dried with filter paper and nitrogen gas. AFM images (10 × 10 μm, 256 × 256-pixel) were acquired on an NT-MDT NTEGRA Prima instrument using tapping mode, a scan frequency of 0.5 Hz, and an NSG01 gold-coated single-crystal silicon probe, with a resonance frequency of ∼150 kHz and a force constant of ∼5.1 N/m. Images were processed using Gwyddion^50^ and analyzed using WSxM software.^51^ In Gwyddion, the images were flattened to the baseline, row aligned with a polynomial degree of 5, and the color range was adjusted for a suitable contrast. In WSxM, the images were flattened, and the lengths and heights of the fibrils were measured using the profile tool. Fibril lengths and height distributions were plotted as histograms. The fibril length distributions were further assessed by fitting Weibull functions as a way to analyze the observed distributions in relation to underlying growth and breakage behaviors, as described by others.^52–54^

### Fibril Cytotoxicity

WT and mutant α-syn fibrils for the cytotoxicity assay were prepared by diluting monomers in sterile DPBS (Gibco. -calcium, -magnesium. #14190144), and the solutions were thereafter passed through 0.22 μm syringe filters to remove potential contaminants. The work was carried out in a laminar flow hood. Fibrils were prepared using the PFF protocol described above. SH-SY5Y human neuroblastoma cells were cultured to confluency in a medium containing 44.5% Ham’s F-12 (Gibco, #11765054), 44.5% MEM, GlutaMAX Supplement (Gibco, #41090036), 10% fetal bovine serum (Gibco, #10099141), and 1% 1× MEM Non-Essential Amino Acids Solution (Gibco, #11140050) in an incubator set at 37 °C, 5% CO_2_. 24 h prior to experiment, the cells were detached and subcultured in the wells of VWR 96-well, flat-bottom, TC-treated cell culture plates (#734-2327) at a density of 20,000 cells/well. Prior to PFF treatments, the cell culture media (CCM) was removed, and the cells were washed 2× with serum-free medium (SFM). Following the wash, PFF samples were diluted to indicated concentrations (0–30 μM) in SFM with 0.01% NaN_3_. To ensure equal salt concentrations across all conditions, additional DPBS was added to all samples except the 30 μM PFF sample before treatment of the cells. After 48 h of treatment, 10 μL of alamarBlue cell viability reagent (Invitrogen, #DAL1025) was added to each well. The resulting alamarBlue emission was read after 3 h of incubation at 37 °C using a BMG Labtech FLUOstar Optima (544 nm excitation, 590 emission filter). All experiments were performed in triplicate (*N* = 3, *n* = 3), and all data were normalized against untreated control, which was set to represent 100% viability. Cytotoxicity was evaluated for statistical significance using two-way ANOVA (*p* < 0.05) with Tukey post hoc tests.

## Results

### Comparison of the Aggregation Kinetics of Wild Type and Mutant α-syn

The aggregation behaviors of wild-type (WT) and mutant variants of human α-syn (Figure 1A) were analyzed using seeded aggregation reactions with 5% nonsonicated preformed fibrils (PFFs) prepared according to the Michael J Fox foundation protocol,^46^ as further described in Section 2. The PFFs were first assessed by AFM (Figure S2), which confirmed their fibrillar nature, and thereafter added to solutions of fresh WT and mutant α-syn monomers under quiescent reaction conditions to form second-generation fibrils (Figure S3). The kinetics of these seeded reactions (monitored by thioflavin-T (ThT) fluorescence)^55^ differed significantly between variants (Figure 1B). For example, WT and H50Q exhibited the fastest growth, reaching a plateau after 40–50 h, whereas A53T was equally fast in the initial growth phase but then proceeded with slower kinetics approaching asymptotically a plateau after >160 h. E46K aggregated slower than these variants, but faster than A30P. A30P, additionally, displayed a biphasic growth behavior. This resembles previously reported aggregation of this variant at low ionic strengths and has been attributed to distinct oligomeric profiles formed during early phases of aggregation.^56^ All variants except A30P displayed concave kinetics without discernible lag phases. This suggests that elongation, rather than secondary nucleation, was the dominant contributor to fibril growth,^40^ as also observed by Buell et al.^27^ for the aggregation of WT α-syn in the presence of equal concentrations of seeds (5%). Notably, the observed differences in aggregation rates among the variants could not be explained by inherent differences in the size of the PFF seeds (Figure S2), suggesting that the available concentration of fibril ends was not rate-limiting in the reactions.

End-point ThT intensities differed significantly among the α-syn variants (Figure 1C, one-way ANOVA, *p* ≤0.0001, Figure S10A). For example, A53T samples exhibited ∼5 times brighter ThT intensities than WT samples at identical starting concentrations of both protein and ThT. To determine fibril yields, we next separated end-point samples into soluble and fibrillar fractions using a previously established centrifugation protocol.^47,48^ The supernatants did not contain any notable fractions of oligomers (Figure S5) and did not produce ThT fluorescence (Figure S6), suggesting that they consisted mainly of monomers. Equally, oligomers or larger amorphous aggregates were not observed in AFM (Figure S3), supporting the conclusion that the absolute majority of aggregated species in our samples were amyloid fibrils. Based on this, we calculated residual monomer concentrations from tyrosine absorption spectra (Figure S4) and used this data to estimate fibril yields. We found that the residual monomer concentrations, and hence fibril yields, varied substantially among the different α-syn variants (Figure 1D) and that the observed differences were statistically significant (one-way ANOVA, *p* = 0.00357, Figure S10B). Notably, ca. 80% of the WT and H50Q monomers but merely 50% of A30P and A53T and 30% of the E46K monomers converted into pelletable amyloid fibrils. For WT α-syn, such yields fall well within range of previously published values.^56–58^ Our data thus suggest that WT and mutant α-syn form amyloid fibrils not only with different kinetics but also with different monomer-to-fibril conversion efficiencies. We performed the same analysis to the original PFF samples (which were prepared using shaking) and found that they had formed with similar yields (Figure S7). Importantly, there were no correlations between the end-point ThT intensities and the fibril yields of the different variants (Figure 1E, Pearson′s correlation test, *r* = −0.17, Figure S10C). This suggests that the reported ThT intensities reflect structural differences rather than fibril concentration variations. We have made similar observations in a previous study on amyloid fibrils formed by different amyloid-β peptides.^55^

To further compare the aggregated α-syn variants, we examined their cytotoxicity in SH-SY5Y human neuroblastoma cell cultures using the alamarBlue metabolic assay. All samples were sonicated prior to cell treatment to fragment the fibrils to a mean size of ca. 150 nm (Figure S8) which eliminates the possibility that differences in toxicity among the fibril variants would relate to their size^59,60^ rather than to structural properties. We found that all α-syn samples produced statistically significant and concentration-dependent reductions in alamarBlue conversion (Figures S9A and S10D) and confirmed that the resazurin/resorufin reagents of the assay did not interact with α-syn fibrils in any way that distorts assay readout (Figure S9B). The observed effects translate into ca. 10–25% decreases in cell viability after 48 h treatment with 30 μM aggregated α-syn (Figure 1F and Figure S9A), which suggest that the fibrils have small-to-modest cytotoxic effects. Statistical testing (two-way ANOVA, *p* = 0.000175, Figure S10D) indicated variant-specific effects on cytotoxicity, but subsequent posthoc multiple comparison (Tukey, Figure S10E) suggests that the differences are rather weak.

### Fibril Fragmentation Alters the Kinetic Trajectories of α-syn Aggregation

Having established that WT and mutant variants of α-syn form amyloid fibrils with differing kinetics and yields (Figure 1), we set up a new seeded aggregation experiment with 50 μM monomers and 2.5 μM (∼5%) seeds (Figure 2). When these reactions (denoted A1 in Figure 2) had reached the stationary phase, we withdrew samples from the well plate and subjected them to ultrasonication to fragment the fibrils and create a greater number of fibril ends. We argued that this could enhance rates of monomer-fibril exchange as observed in hydrogen/deuterium exchange experiments with other amyloid proteins^61^ and hence potentially alter the aggregation trajectories. We have, moreover, observed that WT and E46K α-syn fibrils depolymerized faster in response to cold denaturation after having been subjected to sonication (Figure S11), emphasizing the role of molecular recycling at fibril ends. After the samples had been sonicated, we returned them to the well plate, ensuring the redispensing of equal reaction volumes (see Section 2 for details of the procedure) and thereafter continued to monitor their ThT fluorescence (Figure 2). The cycle of reaggregation (A2–4) and sonication (S1–3), without supplementing the reactions with any additional monomeric or fibrillar α-syn, was repeated several times as schematically depicted in Figure 2A. For reference, we kept replicates of each variant in the well plate throughout the experiment (denoted as "continuous" aggregation in Figure 2). The entire experiment was repeated on three separate occasions, yielding consistent outcomes (Figure 2B–F and Figure S13).

**Figure 1:**
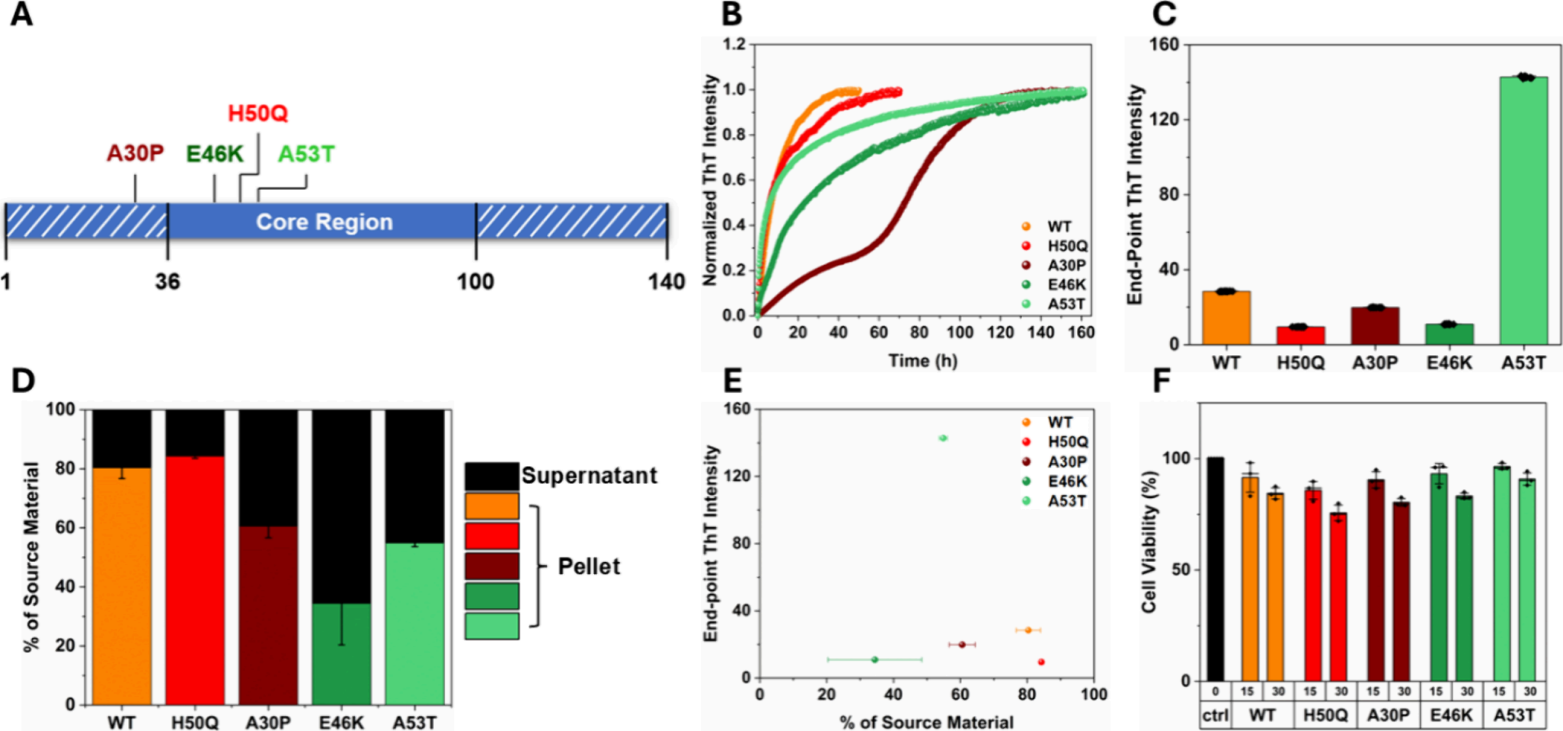
Formation of WT and mutant α-syn preformed fibrils (PFFs) from seeds. (A) Linear representation of the α-syn primary sequence, with the positions of the relevant point mutations in this study marked, as well as the most commonly reported segment of ordered residues in α-syn amyloid fibrilstructures. (B) ThT-monitored kinetics of WT and mutant α-syn aggregation in the presence of 5% PFF seeds. (C) End-point ThT intensities of the kinetic traces shown in (B) differ significantly (one-way ANOVA, *p* < 0.0001; see Figure S10A). (D) Percentage of α-syn (% of source material) in the pellet and supernatant following centrifugation of the samples at the end of the aggregation experiment depicted in (B). One-way ANOVA, *p* < 0.0036 (Figure S10B), shows significant differences in fibril yields between the variants. Error bars represent standard deviation (*n* = 2). (E) End-point ThT intensity plotted against % of source material. Pearson’s correlation test (Figure S10C, PCC = −0.17) shows no correlation between fibril yield and ThT intensity. (F) Cytotoxicity of WT and mutant α-syn PFFs (fragmented to a mean size of ca. 150 nm, Figure S8) measured by alamarBlue fluorescence to probe metabolic activity in cultures of SH-SY5Y cells following 48 h continuous PFF incubation. Two-way ANOVA shows significant effects of both variant (*p* < 0.001) and PFF concentration (*p* < 0.0001) (Figure S10D), whereas Tukey post hoc tests shows that only some cytotoxicity means differed between variants at the 0.05 significance level (Figure S10E). Error bars represent standard deviation of biological replicates (*N* = 3, *n* = 3).

**Figure 2:**
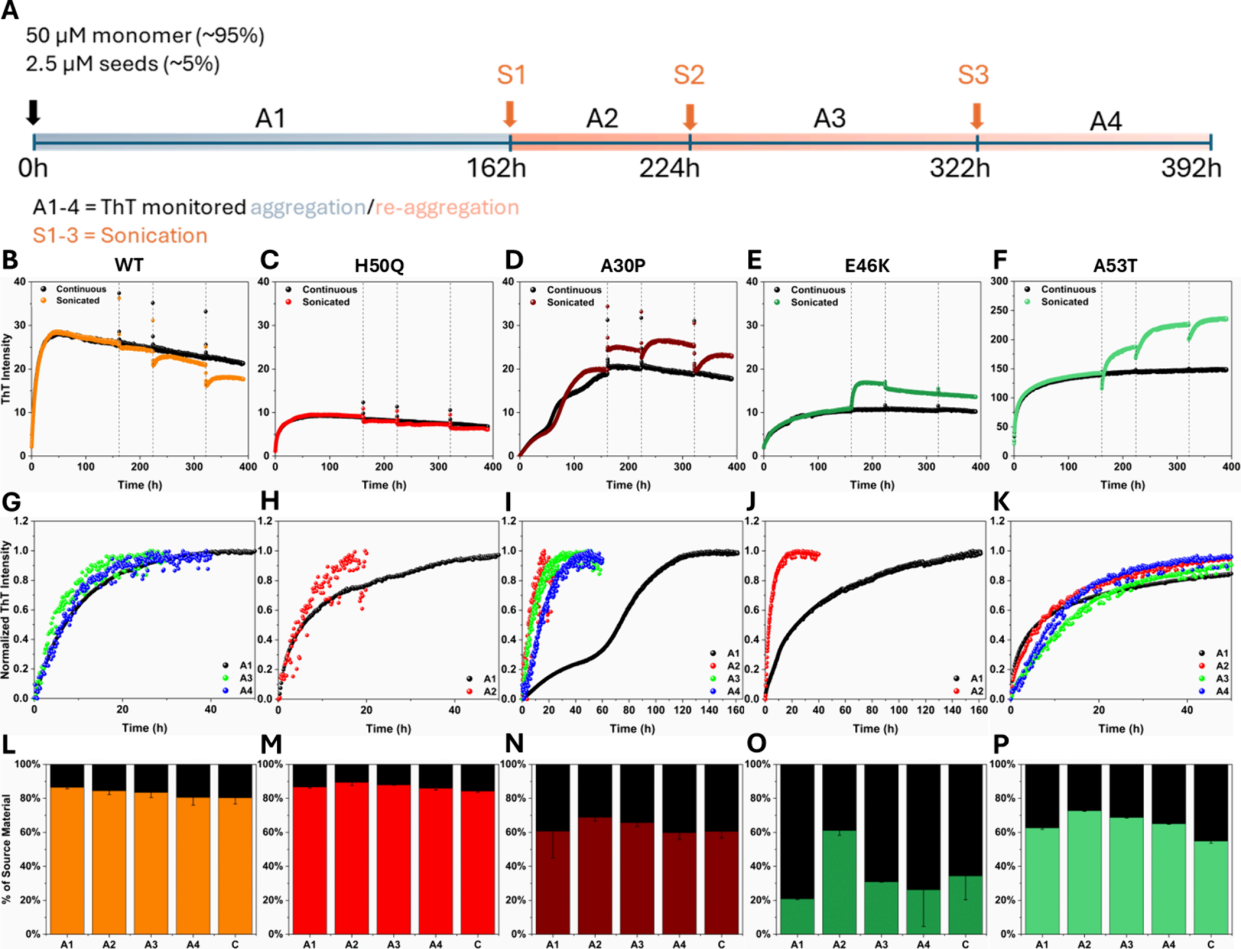
Fibril fragmentation and reaggregation in WT and mutant α-syn samples. (A) Schematic depiction of the experimental setup and the timeline. Solutions of WT and mutant α-syn were subjected to several rounds of aggregation (A1–A4) and sonication (S1–S3) to fragment fibrils, which introduces more fibril ends without changing the total α-syn concentration in the sample. Samples for absorption spectroscopy, SDS-PAGE, and AFM analyses were taken before each sonication step. Additional samples for AFM analysis were also taken after each sonication step. The experiment was repeated three times with one representative experimental round shown. Repeat experiments are shown in Figure S13. (B–F) Aggregation kinetic curves for WT and mutant α-syn monitored by ThT fluorescence. The colored curves represent the samples that were sonicated at time points indicated by the dotted vertical lines in each graph; the black curves represent control samples that were continuously incubated across the entire experiment time. (G–K) Comparison of the aggregation rate of WT or mutant α-syn across aggregations A1–A4 shown as normalized ThT fluorescence. (L–P) Distribution of WT or mutant α-syn (expressed as % of source material) between the pellet and supernatant fractions at the end point of aggregation A1–A4. The samples denoted ‘C’ correspond to the aggregation end-point of the continuously aggregated control. Error bars represent standard deviation (*n* = 2). All samples contained 50 μM monomeric WT or mutant α-syn, 2.5 μM WT or mutant α-syn PFFs, and 20 μM ThT in a 20 mM Tris–HCl buffer at pH 7.4. Aggregation was monitored at 37 °C. No additional monomers were added to the samples after the start of the experiment.

The ThT trajectories of the repeated aggregation-fragmentation experiment are shown in Figure 2B–2F. The time points for sonication are indicated as vertically dotted lines. In most cases, sonication resulted in reaggregation, which typically started from a lower ThT value than immediately prior to sonication (see further below). This supports the hypothesis that sonication results in fibril disassembly and a transient increase in monomer concentration in the samples, which then drives a slower phase of fibril regrowth. To further compare the kinetics in each growth phase, we normalized and overlaid the data for each α-syn variant through A1–A4 (omitting curves with no clear exponential ThT fluorescence increase) (Figure 2G–2K). This showed that the first aggregation reaction differed from the subsequent reaggregations as will be further discussed in the sections below.

The α-syn variants, furthermore, responded differently to the sonication/reaggregation cycles. WT and H50Q essentially followed the trend of their corresponding continuous aggregation curves with the ThT intensity returning to the level of the continuously aggregated sample after each sonication (monomer dissociation and reaggregation) (Figure 2B,C). Their kinetics of reaggregation were somewhat faster than the first seeded step (Figure 2G,H). WT, H50Q, A30P, and E46K α-syn samples displayed gradual declines in ThT fluorescence intensity after the plateau had been reached. We excluded that this decline resulted from ThT photobleaching (Figure S12) and suggest instead that it may be related to excitation/emission loss and/or reduced ThT binding due to lateral association or clustering of the fibrils.^62^ A30P aggregated substantially faster during reaggregation compared to the first seeded step (Figure 2D), the two-step transition that occurred in the initial aggregation phase (Figures 1C and 2D) disappeared, and the ThT intensity increased by approximately 34% (Figure 2D). Most of this intensity increase happened during the first reaggregation (A2). E46K showed rapid reaggregation following sonication S1 and a concomitant intensity increase of approximately 55% (Figure 2E,J) but no further signs of dissociation/reaggregation in the later parts of the experiment. For A53T, there was clear reaggregation occuring after all three sonication steps, all resulting in substantial ThT intensity increase. At the end of the experiment, the ThT intensity for the sonicated A53T samples had increased by 58% compared to the continuously aggregated control (Figure 2F). In contrast to the other α-syn variants, A53T showed slower reaggregation rates compared to the first aggregation phase (Figure 2K).

In addition to monitoring the kinetics, we withdrew 200–300 μL of the samples at each aggregation end point for further analysis. The collected samples were first separated into soluble and pelletable fractions to determine the residual monomer content as described above. The soluble fractions were also analyzed for residual monomer content using SDS-PAGE (Figure S14, Figure S15). Lack of ThT fluorescence was, again, used to confirm the absence of aggregates in the supernatants (Figure S15). However, in this setup, E46K showed a progressive increase in ThT fluorescence intensity in the supernatants during the reaggregation steps (A2–A4), suggesting that sonication of this variant produced ThT-positive aggregates that were too small to be pelleted by centrifugation. The data show that fibril yields remained constant, or near constant, across all steps of sonication and reaggregation (Figure 2L–P), even though the end-point ThT intensities changed for several of the variants (A30P, E46K, and A53T). This behavior is consistent with recent descriptions of monomer solubility in amyloid systems^64^ and suggests that the steady-state conversion of α-syn monomers into fibrils (e.g., the fibril yield) that we observe is, generally, not dependent on the number of available fibril ends and hence not altered by fibril fragmentation. This, furthermore, implies that the observed increases in end-point ThT intensities following sonication/reaggregation must be caused by some structural change in the samples, for example an enrichment or maturation of fibril polymorphs of a type that bind more ThT and/or provide binding sites in which ThT has higher quantum yield; both factors could contribute to stronger fluorescence.^55,65^ The A53T variant, with its continuous increase in ThT emission through all steps of reaggregation stands out conspicuously in this regard.

The results for the E46K variant somewhat contrast this conclusion since we observed a drastic reduction in residual monomer content after the first reaggregation step (A2, Figure 2O) and a concomitant large increase in ThT fluorescence. (Figure 2E). This trend was reproducible across all three independent experiments (Figure S13) and consistent with monomer band intensities in SDS-PAGE (Figure S14). Thus, even though it was not possible to entirely separate monomeric and aggregated E46K by centrifugation, there appears to be a true shift in the monomer-fibril equilibrium for this variant after the first sonication step.

### Sonication and Reaggregation Alter α-syn Fibril Morphology

Having observed that sonication introduced disassembly and reaggregation behaviors in WT and mutant variants of α-syn that were not associated with any changes to fibril mass (Figure 2) but nevertheless, in several cases, resulted in enhancement of ThT fluorescence, we next used AFM to explore possible major evolution of fibril morphologies (lengths and heights) in the samples and across the four aggregation steps (A1–A4) (Figure 3). As noted above, all α-syn variants formed linear filament structures typical for amyloid fibrils, and all samples, even those formed from E46K, were devoid of oligomers. After the seeded aggregation step (A1), some morphological differences were noted. For example, WT and A30P formed long and straight filaments (indicating high persistence lengths),^66^ which were clearly discernible on the mica as individual entities (Figure 3A,C), while H50Q fibrils appeared generally shorter and with a somewhat higher tendency to cluster (Figure 3B). E46K fibrils appeared comparatively more flexible and with a curvilinear shape (Figure 3D) that has commonly been associated with protofibrils,^67^ whereas A53T fibrils were conspicuously short (Figure 3E). It was also more difficult to enrich the A53T fibrils on the mica, which could indicate that they differ in fibril surface properties compared to other variants.^68^

**Figure 3:**
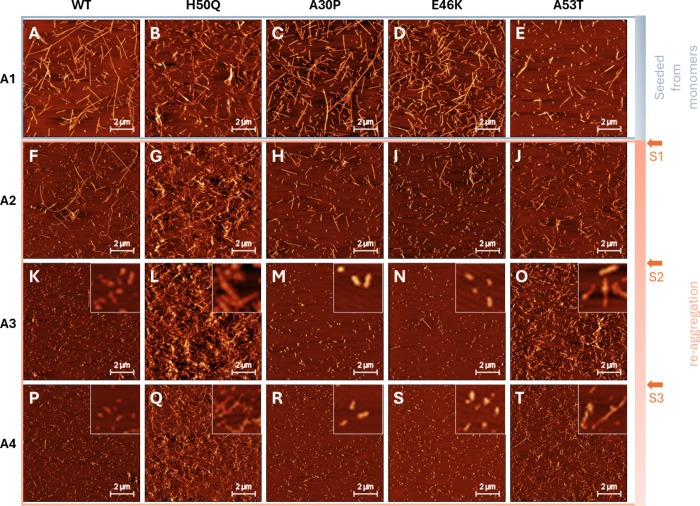
Morphology of WT and mutant α-syn fibrils at the end points of aggregation A1–A4 (as defined in Figure 2). (A–E) AFM images of all fibril variants after the first round of aggregation (A1). (F–T) AFM images of all fibril variants following reaggregation A2-A4. Zoomed-in insets in panels (K–T) are intended to enhance the visual interpretation of fibril structure. All images represent 10 × 10 μm assayed areas, as depicted by the 2 μm scale bars.

Sonication expectedly resulted in the formation of shorter fibrils, and the size reduction of the fibril population remained after reaggregation (Figure 3F–T, denoted A2–A4, orange frame), which is consistent with an increase in fibril numbers. This results from the limited availability of monomers to the increased number of fibril ends. For H50Q, sonication increased fibril clustering to the point that it was challenging to observe single fibrils on the mica despite optimization of sample preparation (Figure 3G,L,Q). Interestingly, the A53T fibrils appeared to stick progressively better to the mica following each sonication and reaggregation round (Figure 3E,J,O,T). This is in line with the above hypothesis that reaggregation may alter structural features of the fibrils, for example through enrichment of specific fibril polymorphs. Furthermore, the increased affinity to mica suggests that, in the case of A53T, which also has the most conspicuous reaggregation behavior (Figure 2), structural change may also manifest in altered chemistry of the fibril surface.

The AFM data was further analyzed to extract α-syn fibril lengths (Figure 4A–E) and fibril heights (Figure 5A–E). This quantitatively supports the observed shortening shown in Figure 3. Furthermore, all α-syn variants formed fibrils with a broad size distribution during the seeded reactions (A1). A53T fibrils were significantly shorter (average of ∼350 nm) than the fibrils of all the other variants (average of ∼600–800 nm). The fibrils thereafter progressively shortened, and their size distributions narrowed, which is consistent with our previous reports of WT α-syn fibril sonication.^59^ We fitted Weibull functions (SI text) to the data in Figure 4 to further analyze fibril length distributions, as previously described by Xue et al.^52^ and Iadanza et al.^69^ The distributions explain data reasonably in most cases, albeit with A53T being an exception possibly indicating differences in its growth and fragmentation behaviors. Furthermore, by analyzing the Weibull distribution shape parameter κ (Figure S16B), we suggest that the initial fibril length distributions (in A1) are consistent with apparent random growth and breakage (κ-values close to 1), whereas the increase in κ with number of sonications suggests that as fibrils shorten, the fibril breakage propensity becomes increasingly dependent on fibril length, as also suggested by others.^52,70^

**Figure 4:**
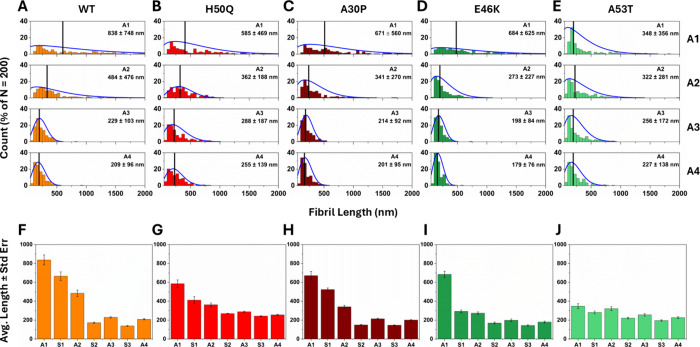
Length distributions and mean lengths of WT and mutant α-syn fibrils. (A–E) Fibril lengths were extracted from AFM images of the type in Figure 3, which were taken at the end point of each aggregation (A1–A4), as defined in Figure 2. The black lines in (A–E) represent the mean length. The blue curves in (A–E) represent Weibull distributions fitted to the data. The shape (κ) and scale (λ) parameters of the Weibull distributions are shown in Figure S16. One to seven independent AFM images were analysed, counting in total 200 fibrils (*n* = 200) per sample. (F–J) Mean fibril length ± standard error (*n* = 200) in reactions A1–A4, alongside time points S1–S3, which represent samples taken for AFM analysis immediately after the sonication step.

**Figure 5:**
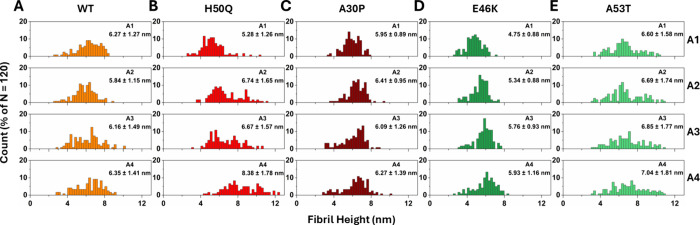
Height distributions of WT and mutant α-syn fibrils. Fibril heights for (A) WT, (B) H50Q, (C) A30P, (D) E46K, and (E) A53T were extracted from AFM images of the type shown in Figure 4, which were taken at the end point of each aggregation (A1–A4), as defined in Figure 2. One to seven independent images per condition were analysed, counting in total 120 fibrils (*n* = 120) per sample.

In addition to comparing fibril lengths at the end point of each aggregation phase (immediately before sonication), we collected samples immediately after each sonication step (Figure S17). This showed that the average fibril length of each of the different α-syn variants converged to a lowest value and that the fibril population thereafter oscillated between a sonication-induced shortening and a re-aggregation induced extension (Figure 4F–J). This is consistent with the notion that fibrils will not break below a certain length cutoff and with the above conclusion that fibril breakage becomes less frequent with decreasing fibril size. We found that all fibril types, except A53T, shortened substantially during S1 and S2. Thereafter, the effect was much smaller, suggesting that the fibrils had reached the shortest achievable distribution with set sonication force.

Interestingly, we observed a decrease in average fibril length during the first reaggregation step (A2) for all variants except A53T. This could for example be explained by fibrils continuing to depolymerize in these samples following sonication in combination with continued high secondary nucleation rates such that the formation of new fibril species is favored over the elongation of existing ones. In subsequent reaggregation steps (A3–A4) and all reaggregation steps for A53T, there was an increase in fibril length following sonication, suggesting that existing fibrils grew mainly via elongation. At the later stages of the experiment, the average fibril lengths were found to oscillate between a minimum (which is likely dictated by the sonication conditions) and a maximum (which is likely dictated by re-establishment of the steady state in the system). Interestingly, the minimum average lengths adopted by the different α-syn variants differed, despite the fact that all fibril samples were subjected to the same sonication force. The H50Q and A53T fibrils were longer than fibrils of other variants.

Lastly, we used the AFM data to analyze fibril heights (Figure 5) and explored the possible coexistence or evolution of fibril polymorphs with major differences in protofilament assembly. We found that all fibrils that had formed after the first seeded aggregation (A1) had average heights of ∼5–6 nm (Figure 5A–E, top row). This is consistent with published structural models of α-syn fibrils with two intertwined protofilaments,^71^ which also appears to be the most common α-syn structure. All variants except A53T had fibril heights that were relatively homogeneously distributed around a single maximum, whereas A53T (Figure 5E) appeared to have an additional small fibril population with larger height. This could suggest a higher degree of intrinsic polymorphism in this variant. Following sonication and reaggregation, the height distributions changed for some of the variants. For example, the H50Q fibrils (Figure 5B) increased in average height across all phases of reaggregation, reaching an average of 8.4 nm at the end of the experiment. E46K fibrils (Figure 5D) also gradually increased in height, albeit less drastically. Notably, after the first seeded aggregation step (A1), the E46K fibrils were considerably thinner than all other fibril variants. This, together with their initial curvilinear appearance, suggests that they first assemble into protofibril-like structures that then convert into thicker fibrils. Notably, E46K was the only variant for which the monomer-fibril equilibrium appeared to shift after the first sonication (Figure 2).

### α-syn Fibrils Reaggregate with Different Elongation Rates

To further explore the kinetics of the seeded aggregation and reaggregation steps (Figure 2), we fitted single-exponential functions (SI text) to the aggregation curves (Figure 6A–C and Figure S18). The fit to data was good in all cases (*R* ^2^ > 0.90, Figure 6D), suggesting that the reactions were dominated by fibril elongation,^27^ consistent with the fact that the reactions were strongly seeded. Elongation rate constants (Figure 6E and Figure S18) were then estimated using the time constants from the curve fitting, data on fibril yields (Figure 2), and estimated mean fibril lengths (Figure 4) as detailed in the Supporting Information. The elongation rate constant for seeded WT α-syn aggregation (reaction A1) was 2087 M^–1^ s^–1^, which is in good agreement with previously reported data (2200 M^–1^ s^–1^ ^27^ and 3616 M^–1^ s^–1^ ^72^). We found that the elongation rate constants differed among the α-syn variants. In addition, elongation rates within each variant's samples were generally different in the different aggregation (A1) and reaggregation (A2–A4) steps (Figure 6E), but with no clearly generalizable trend. For example, the elongation rate in WT α-syn decreased with each reaggregation step, whereas E46K and H50Q elongated faster during reaggregation than in the first seeded phase. For A53T, we noted a significant improvement in the exponential fit (Figure 6D) during reaggregation. This, together with the observation that A53T fibrils were generally shorter than other variants prior to the first sonication, suggests a proportionally higher degree of secondary nucleation, alongside elongation, for this variant.

**Figure 6:**
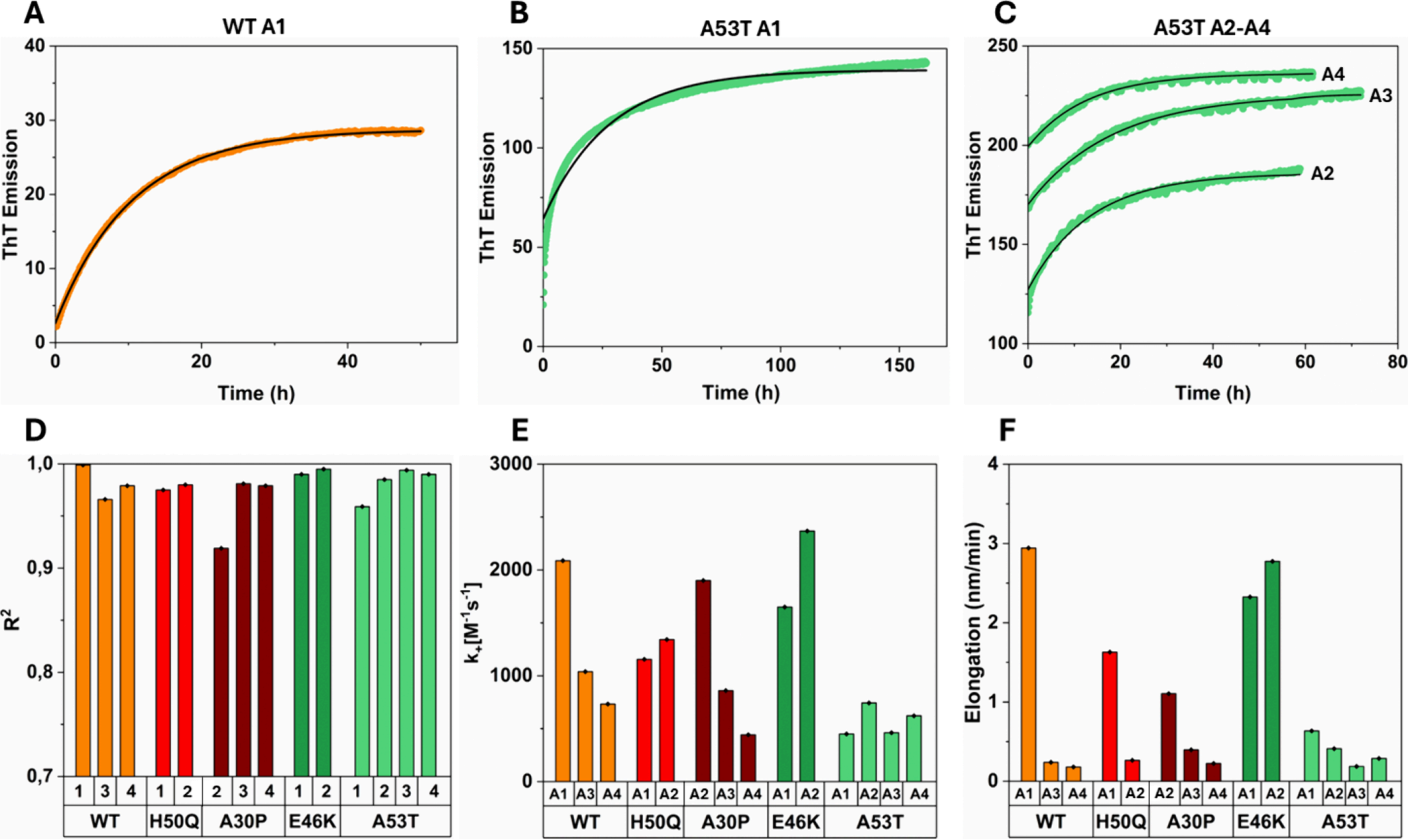
Fibril elongation rates. (A, B) Representative kinetic curves of respectively WT and A53T aggregation in the first aggregation phase (A1), fitted to a single-exponential model describing elongation-dominant fibril growth. (C) Representative kinetic curves of A53T reaggregation (A2–A4), fitted to the same exponential model as in (A, B). Additional fitted kinetic curves are shown in Figure S18. (D) Comparison of goodness of fit (*R* ^2^) of the data in (A–C) and Figure S18. (E) Estimated elongation rate constants (M^–1^ s^–1^) and (F) elongation speed per fibril (nm/min).

We finally also estimated the elongation speed per fibril (e.g., nm growth per minute) (Figure 6F and Figure S18) using the rate constants in Figure 6E and the monomer concentration at the start of each aggregation and reaggregation step (see SI text for details). The elongation speed was generally fastest during the first aggregation step (A1), which was expected given that the monomer availability was high and then reduced in the reaggregation steps when the numbers of available monomers per fibril were considerably lower. E46K, however, had faster elongation in reaggregation A2, which is consistent with our suggestion that the monomer-fibril equilibrium for this variant may actually shift upon sonication and the observation of curvilinear protofibril-like aggregates in step A1 (see above).

## Discussion

Protein aggregation into amyloid fibrils occurs by complex mechanisms that involve multiple reaction steps, including primary and secondary nucleation and fibril elongation.^40^ The fibril assembly has been shown to be highly environment dependent and described as to occur on a rugged energy landscape^73,74^ and largely under kinetic control.^75^ With the rapidly increasing number of available high-resolution amyloid structures, it has become increasingly evident that the same protein can adopt many different amyloid folds, that fibril polymorphs coexist within samples,^76,77^ and that amyloid fibrils formed by, at least some proteins, have the ability to evolve with time.^10,19^ Understanding this complexity, and how different aggregation conditions shape both reaction kinetics and fibril structure, is an important challenge, given the strain behavior of amyloid propagation disease^78^ and reports of distinct fibril polymorphs having distinct toxicities.^79^

In this study, we explored the aggregation of WT and mutant variants of α-syn using conventional ThT-based kinetics, but with the addition of a fibril fragmentation step when the reactions had reached the plateau phase. We report fragmentation to result in an initial rapid fibril disassembly and subsequent reaggregation, through which the monomer-fibril equilibrium of the first reaction phase is reestablished.

Using this modified ThT assay, we found that some of the α-syn variants, conspicuously those associated with early-onset forms of Parkinson’s disease (A30P, E46K, and A53T), successively increased their ThT fluorescence with increasing number of fragmentation steps, despite that the protein concentration was kept constant and without observed changes in fibril mass. This suggests that α-syn, akin to what has recently been shown for IAPP^10^ and tau^19^ using cryo-EM, has the capability to structurally evolve with time. Possibly, such processes are aided by fibril formation occurring on a rugged energy landscape^80^ and the coexistence of kinetically trapped, rather than thermodynamically defined, fibril conformers with similar stabilities. Furthermore, the observation that early-onset variants of α-syn particularly evolved their ThT fluorescence raises the possibility that polymorphic flexibility may be important for early initiation and rapid progression of disease.

Recent research has focused heavily on the important contributions of primary nucleation, elongation, and secondary nucleation to the process of α-syn fibril growth.^81–83^ This study suggests that fragmentation, in addition to enhancing elongation rates, may facilitate, or favor, the formation of certain fibril polymorphs. Although fragmentation occurs with low frequency under quiescent conditions *in vitro*, fibril breaking processes may be more prominent *in vivo*. A primary neuron-based study of Lewy body formation has, for example, shown that α-syn fibrils in inclusions were considerably shorter than those initially formed through exogenous seeding.^84^ *In vivo* fibril fragmentation may result from the activity of molecular chaperones like HSP104 ^85^ and proteolytic enzymes such as the protease Cathepsin D,^86^ but it has also been suggested that α-syn fibrils as such are less stable toward breakage than disease-unrelated fibril types.^87^

Our data suggest that sonication, and the associated fibril fragmentation, increases not only the number of fibrils but also the frequency of disassembly and reassembly at fibril ends. Such recycling of protein monomers has previously been reported to contribute to the dynamics of amyloid fibrils.^61^ Our observation that these processes result in a change to the fibril state in the samples (increased ThT fluorescence) for α-syn variants A30P, E46K, and, particularly, A53T suggests that molecular recycling at fibril ends can further drive enrichment of certain fibril polymorphs. These polymorphs could either be more resistant toward disassembly or exhibit lower energy barriers for elongation or a combination of both.

Amyloid-induced ThT fluorescence results from restricted dissipation of excitation energy through hindered molecular rotation as ThT molecules bind snugly in between the interdigitated protruding side chains of the cross-β amyloid core,^88^ and parallel to the fibril axis.^89^ The magnitude of ThT augmentation depends on several factors, including fibril mass (available binding sites), binding affinity, and ThT fluorescence quantum yields.^65^ The latter two depend on fibril structure and have, for example, been used by us to explain why fibrils of amyloid-β variant Aβ40 fibrils exhibit significantly higher ThT fluorescence than those formed of the variant Aβ42.^55^ Morphological comparison of these related fibril types has further revealed that Aβ40 fibrils, with higher ThT fluorescence, were straight whereas Aβ42 fibrils appeared more curvilinear.^90^ This suggests that high ThT intensity is associated with more well-ordered fibril structures. It is thus possible that the fragmentation-induced increase in ThT intensity that we observed here, particularly for the A53T fibrils, results from an enrichment process in which less ordered fibrils disassemble to provide monomers that can elongate more ordered fibril polymorphs. It should be noted that the macroscopic morphological differences between such polymorphs may be rather subtle.

In addition to observing differences in the fragmentation-induced disassembly/reassembly behavior of WT and mutant α-syn variants, we report substantial differences in residual monomer content in reactions that reached steady state. These results could also be rationalized in relation to the association of different α-syn variants to, respectively, late and early onsets of Parkinson’s disease. Our findings suggest that the early-onset Parkinson’s disease variants A30P, E46K, and A53T can have reduced fibril conversion rates relative to WT. This raises the possibility that these fibrils may either have lower stability or differ in their tendency to adopt kinetically trapped fibril states, alongside their above-discussed increased tendency of molecular recycling and fragmentation-induced structural evolution. It is possible that these early-onset α-syn variants initially assemble via the population of transient and less well-structured fibril species, such as the curvilinear E46K that we observed after the first aggregation step in this study, and that these species somehow have toxic profiles that, despite not manifesting strongly in our simple cytotoxicity assessment, contribute to enhanced neuronal vulnerability and hence earlier disease onset. Such behavior, where fibrils with distinct morphologies exhibit varying stability and toxicity, has been previously shown for α-syn ribbons and twisted fibrils,^79^ prions,^91^ and insulin fibrils.^92^

In this work, the fibril fragmentation resulted in α-syn reaggregation under strongly seeded conditions, and the reactions were hence expected to become increasingly dominated by fibril elongation, as was also verified by the fitting of the kinetic data to a simplistic model of exponential fibril growth. The fitting became progressively better with each reaggregation step for the early-onset variants (A30P, E46K, and A53T), suggesting that secondary nucleation became less and less frequent. In addition, we found that the elongation speed per fibril end progressively decreased, which can be explained by fragmentation resulting in more fibril ends and hence less monomer availability to elongate each fibril. A study comparing Aβ40 and Aβ42 fibrils has suggested that elongation occurs via a "dock-lock" mechanism, where the slower "locking" step involves important conformational rearrangement of the monomer to fit or template the structure at the fibril end.^93^ Our data are consistent with such a model and suggest that the A30P, E46K, and A53T variants of α-syn, when allowed to reassemble by slow elongation, adapt to slow locking and a better replication of orderly fibril structures at the expense of less ordered ones.

In conclusion, we report that α-syn fibrils, particularly those formed by mutants associated with early-onset forms of Parkinson’s disease, are characterized by aggregation reactions that result in relatively high levels of residual monomers. We furthermore show that these variants, more extensively than WT and other late-onset mutants, can structurally evolve in response to fibril fragmentation, such that their fibril populations became characterized by increasingly bright ThT fluorescence. We suggest that this results from a process of monomer disassembly and reassembly at fibril ends. Altogether, our study identifies connections between biophysical and structural parameters of α-syn aggregation, such as their polymorphism and stability, and Parkinson’s disease onset at progression. We furthermore highlight the importance of fragmentation as a mechanism of promoting molecular recycling at fibril ends and templated elongation toward the selection of stable, and putatively less toxic, amyloid polymorphs.

## Supplementary Material



## Data Availability

Data used in the article has been made available through public repository.

## Notes

SEC chromatograms showing monomerization of the different α-syn variants; AFM images showing the morphology of the different α-syn PFFs and of fibrils formed after the first reaggregation; protein absorption and ThT emission spectr; SDS-PAGE gels underlying the analysis of residual monomer content and fibril yield; graphs showing residual monomer content of PFF seeds; average lengths of sonicated fibrils used for cytotoxicity testing; concentration dependence of cell viability; data from statistical tests; graphs showing the decomposition of sonicated and full-length WT and E46K fibrils at low temperature as a means of comparing their stability; data confirming that ThT is not photobleached by the total excitation burden introduced during the long acquisition of kinetic data; graphs showing the reproducibility of the aggregation/reaggregation behavior of WT and mutant variant α-syn, including SDS-PAGE depicting residual monomer content; graphs showing Weibull parameters obtained by fitting fibril length distributions; graphs showing fibril length distributions immediately after sonication; fitting parameters and graphs showing goodness of fit for the estimation of elongation rate constants from kinetic data; and procedures of fitting fibril length distributions and estimating elongation rate constants (PDF)
