# Supplementary material for: Fragmentation-Induced Disassembly and Reaggregation of α‑Synuclein Amyloid Fibrils
Source: ACS Chem Neurosci. 2026 May 20;17(11):2057–70. doi: 10.1021/acschemneuro.5c00895 (PMC13237733; doi:10.1021/acschemneuro.5c00895)
Supplement: Supplementary file 1 [file cn5c00895_si_001.pdf]

Supporting Information to

Fragmentation-induced disassembly and reaggregation of α-synuclein amyloid fibrils

*Fritjof Havemeister, Vesa Halipi, Marziyeh Ghaeidamini, and Elin K. Esbjörner**

Division of Chemical Biology, Department of Life Sciences, Chalmers University of Technology, Kemivägen 10, 412 96 Gothenburg, Sweden

* Corresponding author: E.K.E. eline@chalmers.se. Phone +46 31 772 5120

1. Supporting data figures
2. Supporting text

1. <u>Supporting Data</u>

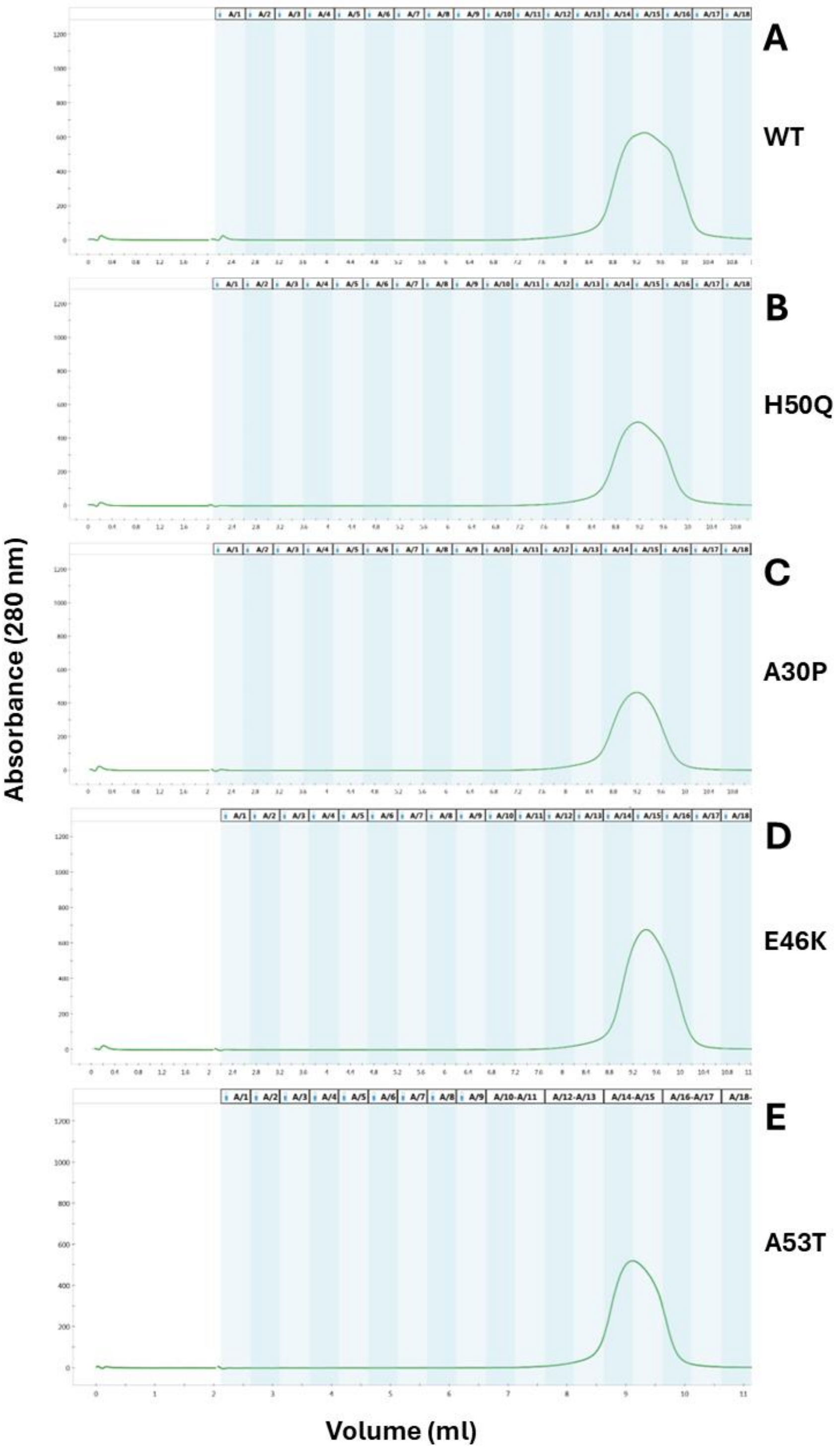

Figure S1. Purification of WT, H50Q, A30P, E46K, A53T α-syn monomers after thawing. All size-exclusion chromatograms show that monomers were eluted as a single peak between 8.5-10.5 ml.

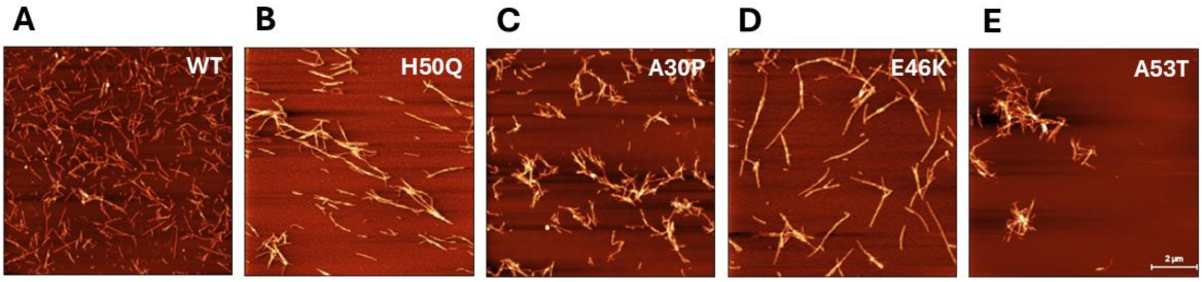

Figure S2. Characterization of PFFs made according to the MJF foundation protocol. (A-F) AFM images of WT, H50Q, A30P, E46K and A53T α-syn PFFs. All images are 10x10 µm as shown by the 2 µm scalebar in (E).

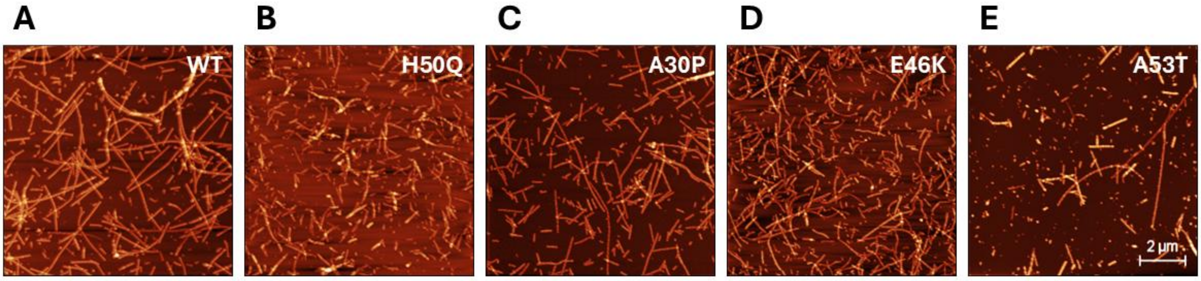

Figure S3. AFM images of fibrils formed after the first re-aggregation (A1). All images are 10x10 µm as shown by the 2 µm scalebar in (E).

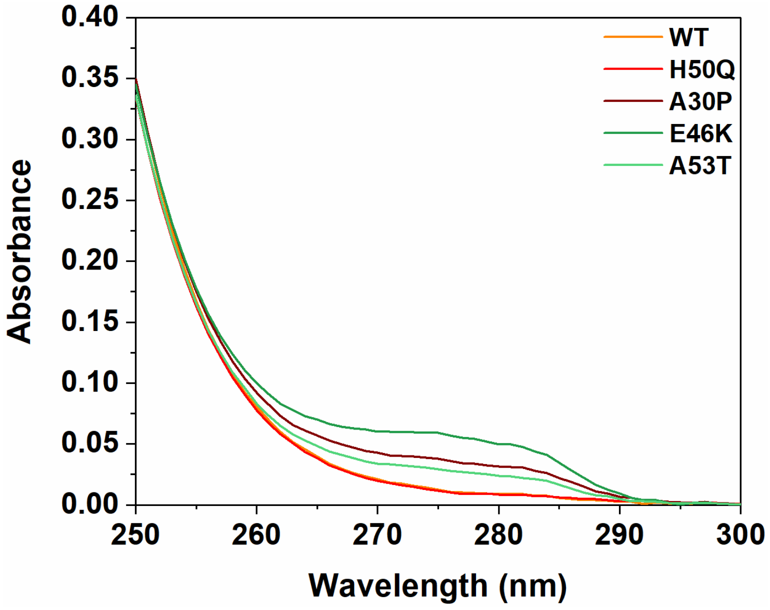

Figure S4. Analysis of residual monomer content in supernatants after aggregation A1 measured using tyrosine absorption.

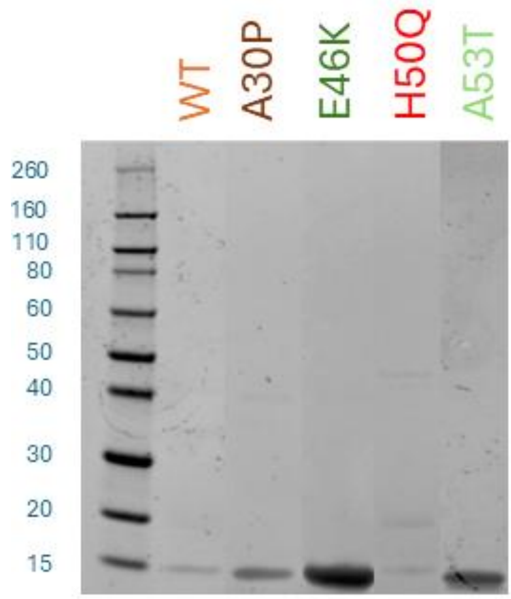

Figure S5. Residual monomer analysis. SDS-PAGE of the supernatants (WT, A30P, E46K, H50Q, A53T) after aggregation A1, showing clear monomer bands and no significant accumulation of higher order oligomers.

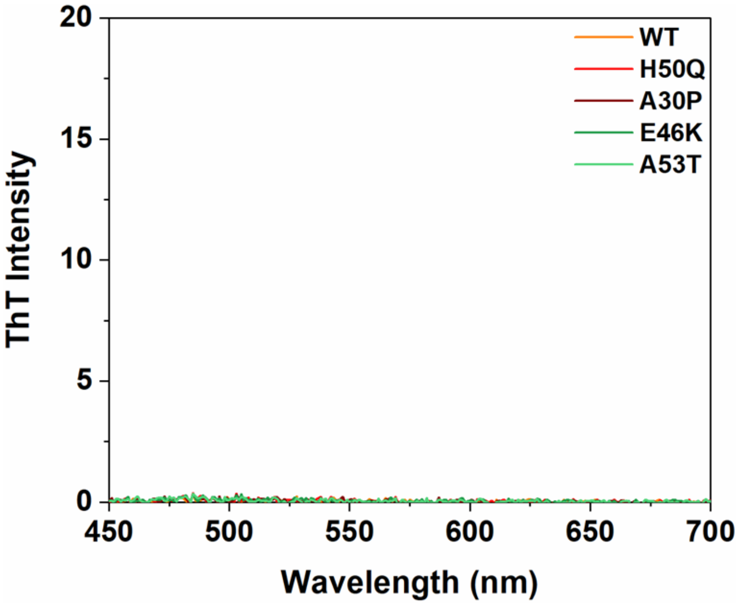

Figure S6. Analysis of putative fibrillar aggregates in supernatant. ThT spectra of WT, H50Q, A30P, E46K, A53T samples after aggregation A1 showing the absence of ThT positive aggregates.

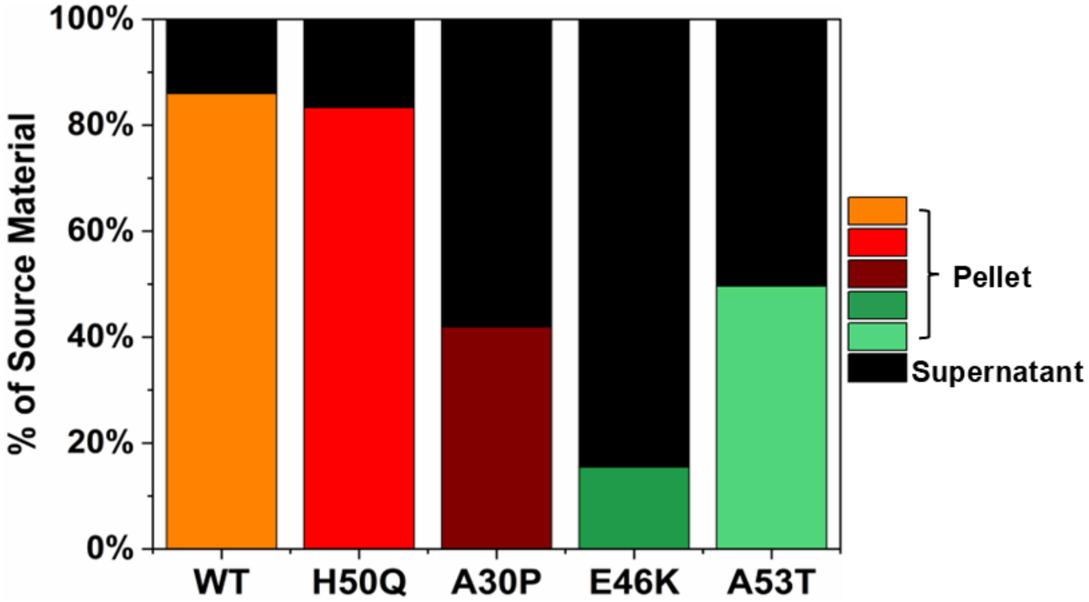

Figure S7. Residual monomer content of PFF seeds made according to the MJF foundation protocol. The residual monomer content was determined based on tyrosine absorption in supernatants.

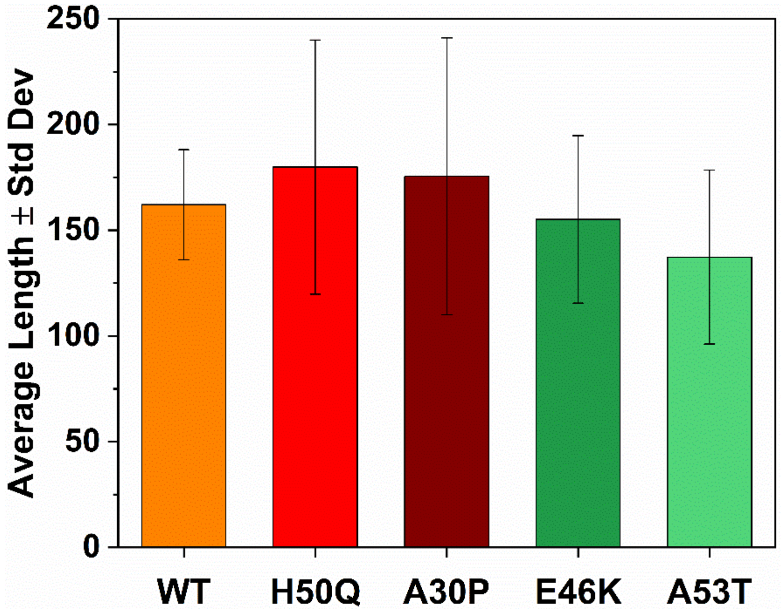

Figure S8. Characterization of fibrils used for cytotoxicity studies in SH-SY5Y neuroblastoma cells after sonication. The length of fibrils were determined by analysis of AFM images and the data show that samples fed to SH-SY5Y cells had a mean fibril size of ~150 nm. (n=200).

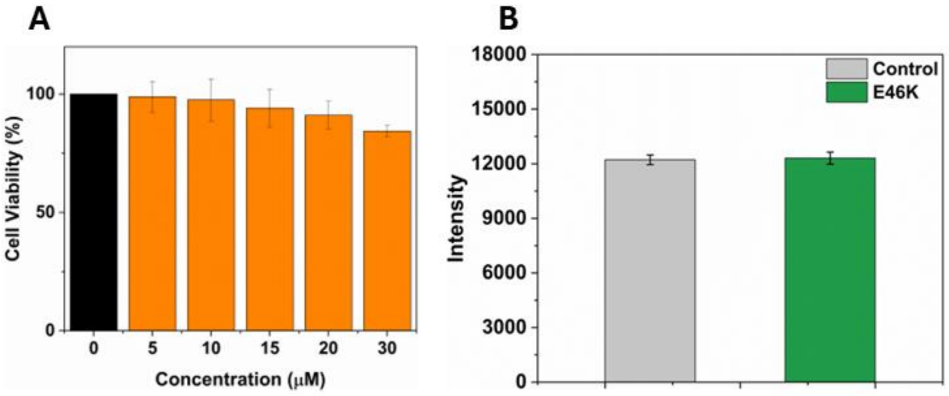

C

	DF	Significance Level	Sum of Squares	Mean Square	F Value	P value	Overall ANOVA
Concentration	5	0,05	694,97	138,99	3,72	0,01728	Significant

Figure S9. (A) Cell viability of SH-SY5Y neuroblastoma cultures treated with increasing concentration gradient of WT α-syn PFFs formed according to MJF protocol. (B) Control experiment verifying that α-synuclein fibrils do not intercalate with Alamar blue as shown by similar fluorescence intensities of Alamar blue incubated with media and incubated with E46K fibrils and media. (C) Oneway ANOVA ($p < 0.05$) indicates significant differences in cytotoxicity across PFF concentrations.

A

	DF	Significance Level	Sum of Squares	Mean Square	F Value	P value	Overall ANOVA
End-Point ThT	4	0,05	1,29E11	3,21E10	618244,74	<0.0001	Significant

B

	DF	Significance Level	Sum of Squares	Mean Square	F Value	P value	Overall ANOVA
Fibril Yield	4	0,05	904,83	226,21	18,05	0,00357	Significant

C

	Nr of Points	DF	Residual Sum of Squares	Pearson's	Correlation
ThT Intensity – Fibril Yield	5	3	41,28	-0,17425	No Correlation

D

	DF	Significance Level	Sum of Squares	Mean Square	F Value	P value	Overall ANOVA
Variant	4	0,05	353,38	88,34	7,93	1,75E-4	Significant
Concentration	2	0,05	2187,07	1093,53	98,10	<0.0001	Significant
Interaction	8	0,05	203,53	25,44	2,28	0,04855	Significant

E

Comparison	Significance Level	P Value	Tukey Test
H50Q - WT	0,05	0,03364	Significant
A30P - WT	0,05	0,82372	Not Significant
A30P - H50Q	0,05	0,28572	Not Significant
E46K - WT	0,05	0,99997	Not Significant
E46K - H50Q	0,05	0,02623	Significant
E46K - A30P	0,05	0,76898	Not Significant
A53T - WT	0,05	0,14113	Not Significant
A53T - H50Q	0,05	<0.0001	Significant
A53T - A30P	0,05	0,01284	Significant
A53T - E46K	0,05	0,17232	Not Significant
15 µM – 0 µM	0,05	<0.0001	Significant
30 µM – 0 µM	0,05	<0.0001	Significant
30 µM – 15 µM	0,05	<0.0001	Significant

Tables S10. Statistical test parameters for data in Fig. 1C-F in the main text. (A) Significant differences in end-point ThT intensity between the variants (One-Way ANOVA). (B) Significant differences in fibril yield between the variants (One-Way ANOVA). (C) No correlation between the end-point ThT intensity and the fibril yield (Pearson's Correlation). (D) Fibril cytotoxicity was significantly dependent on concentration and differed significantly among variants. The interaction between the factors was also significant (Two-Way ANOVA). (E) Tukey post hoc test following the Two-Way ANOVA in (D) showed that the cytotoxicity means were only significantly difference when comparing some of the variants. A threshold

level of $p=0.05$ was set in all tests, data tables show actual p-values obtained. Statistical testing was performed using Origin software.

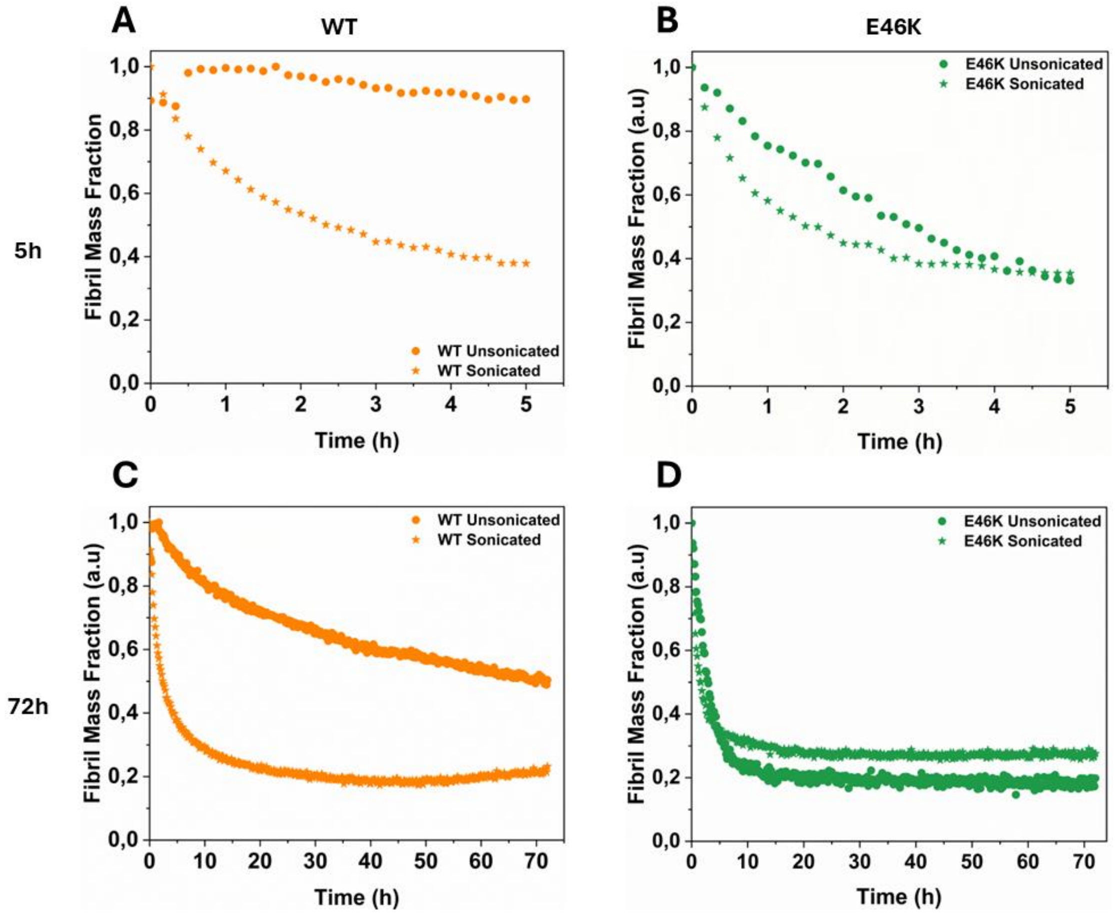

Figure S11. Cold denaturation of WT and E46K fibrils. (A-B) Decomposition of unsonicated and sonicated WT and E46K fibrils under cold denaturation. Data were acquired using CD spectroscopy, monitoring the loss of β-sheet signature at 2018 nm and then converted into fibrillar mass fractions. (C-D) Decomposition of unsonicated and sonicated WT and E46K fibrils under cold denaturation across 72 hours of incubation, measured as described above. The cold denaturation temperature was set to 4ºC, and data were acquired under gentle stirring conditions to avoid sample sedimentation.

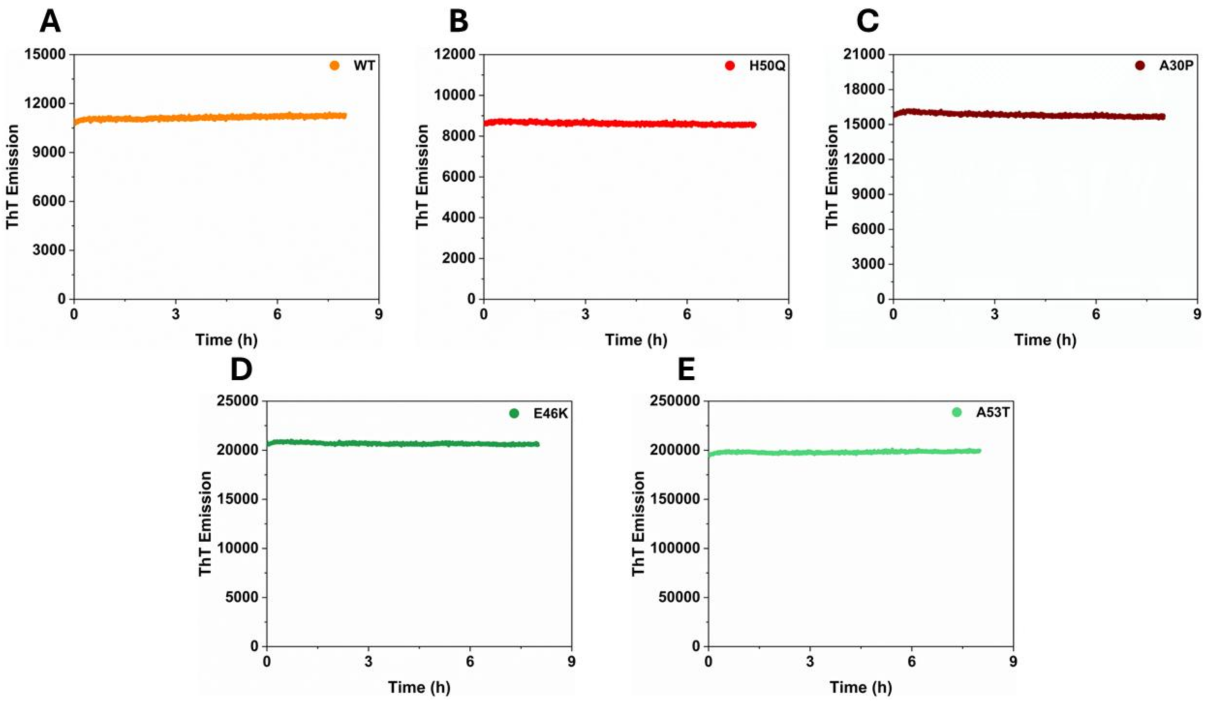

Figure S12. Control showing lack of ThT photobleaching in the kinetic assay. WT, H50Q, A30P, E46K, and A53T α-syn fibrils (52.5 μM) were mixed with 20 μM ThT and distributed in 96-well plates at corresponding volumes as in the kinetic experiments. The samples were subjected to the same total excitation light burden as in the full kinetic experiments in Fig. 2 of the main text, but over a shorter time period to avoid potential fibril sedimentation, clustering, maturation, or other interactions that may influence the ThT read-out. The samples were excited at 12-second intervals over 2,400 cycles, matching the total number of excitations and total light exposure in Fig. 2B-2F.

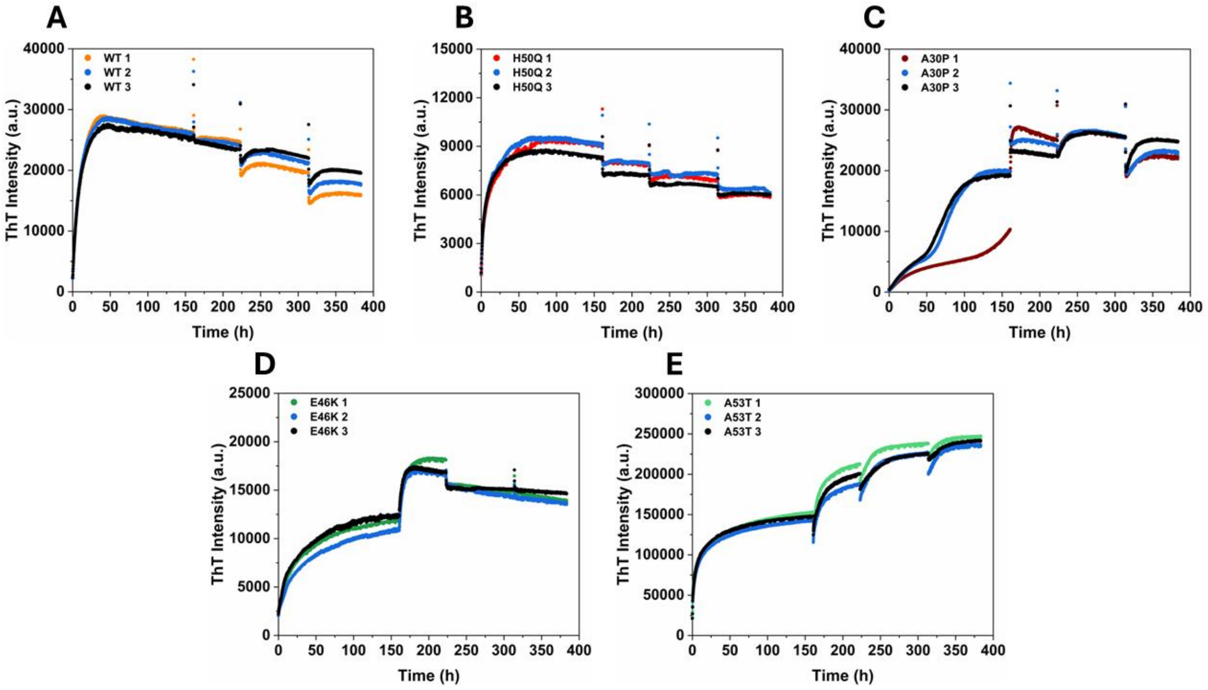

Figure S13. Overlay of three independent experiments of the aggregation and re-aggregation kinetics of WT and mutant α-syn, complementing the data shown in Fig. 2 of the main text, demonstrating the reproducibility in aggregation and re-aggregation behavior. (A) WT, (B) H50Q, (C) A30P, (D) E46K, (E) A53T.

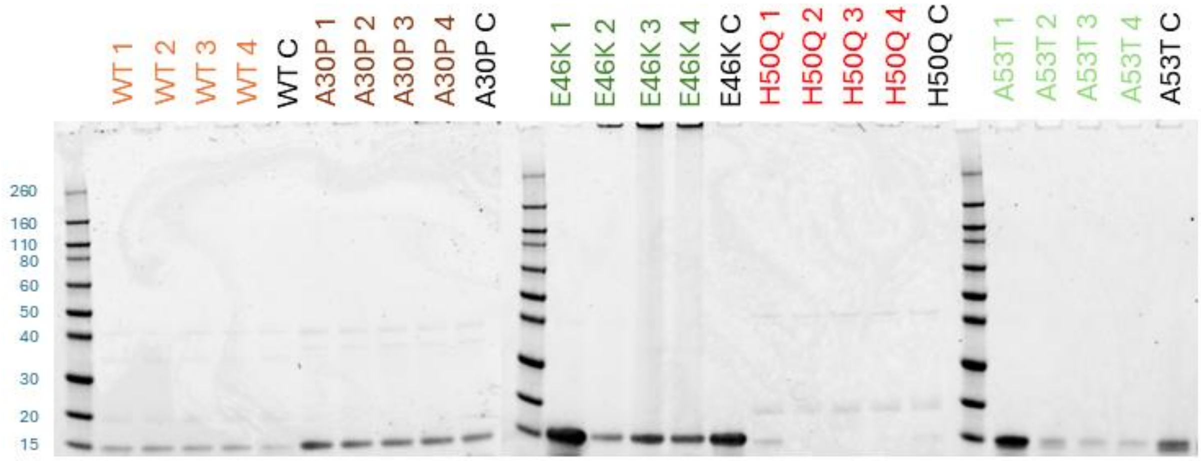

Figure S14. Residual monomer content visualized on SDS-PAGE. SDS-PAGE demonstrating the residual monomer content for every aggregation (A1-A4) and variant (WT, H50Q, A30P, E46K, A53T).

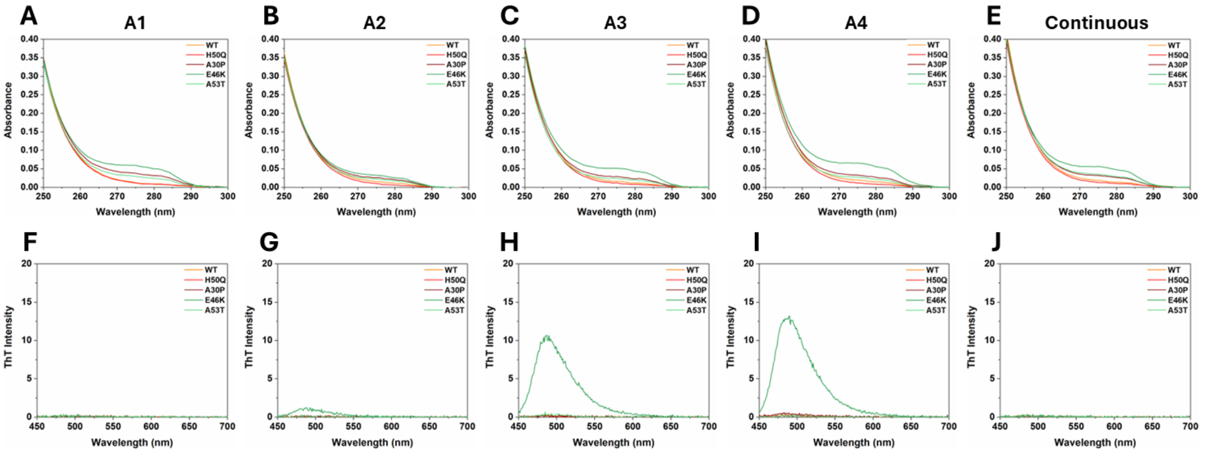

Figure S15. Absorbance and ThT fluorescence spectra underlying the analysis of fibril yield in main text Fig. 2L-2P. The spectra were recorded on supernatants from centrifuged samples collected at the end-point of phases A1 to A4 (see main text Fig. 2L-2P for figure annotation) or at the end-point of a continuously incubated sample(‘continuous’). (A-E) tyrosine absorption in supernatants used for quantification of soluble fraction (F-G) ThT fluorescence tests, verifying that the supernatants, in all cases except E46K, were free of ThT positive fibrils.

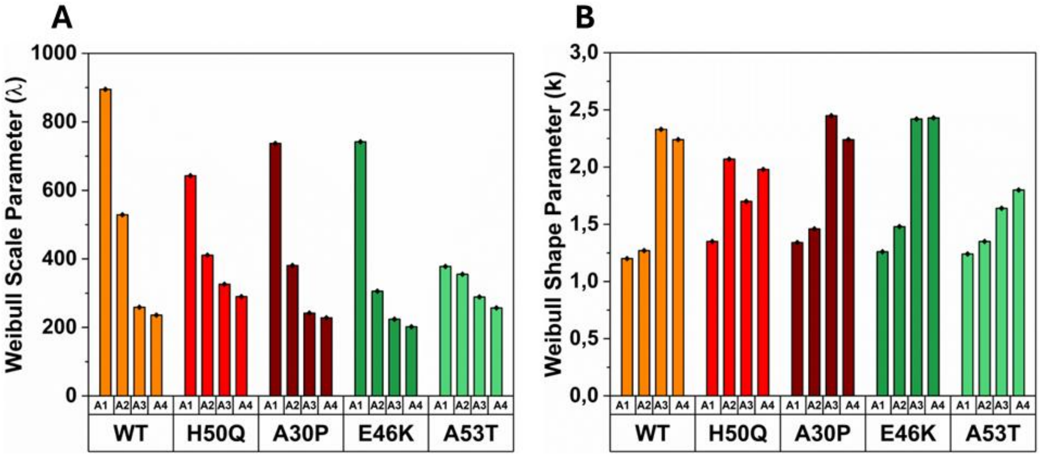

Figure S16. Weibull distribution parameters from Fig. 4 (A-E). (A) Weibull scale parameter (λ) showing the characteristic fibril length and (B) the Weibull shape parameter (k) showing the length-dependence of fragmentation for each variant and in each aggregation A1-A4. A k > 1 indicates that longer fibrils are more prone to fragmentation than shorter ones, and an increasing k (A1-A4) shows that fragmentation becomes progressively more selective toward breaking longer fibrils.

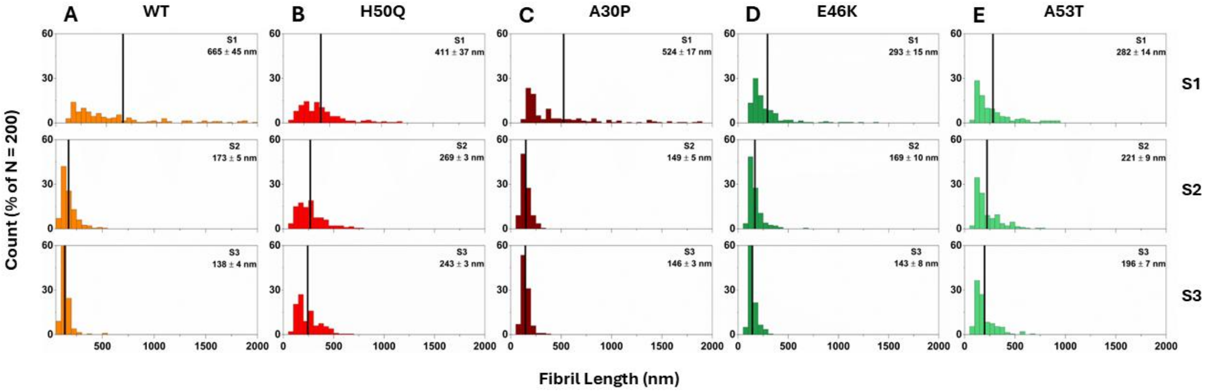

Figure S17. Length distributions of fibrils immediately after sonication S1-S3. (A) WT. (B) H50Q. (C) A30P. (D) E46K. (E) A53T. Black bars represent the mean length of the fibrils. n=200 per condition.

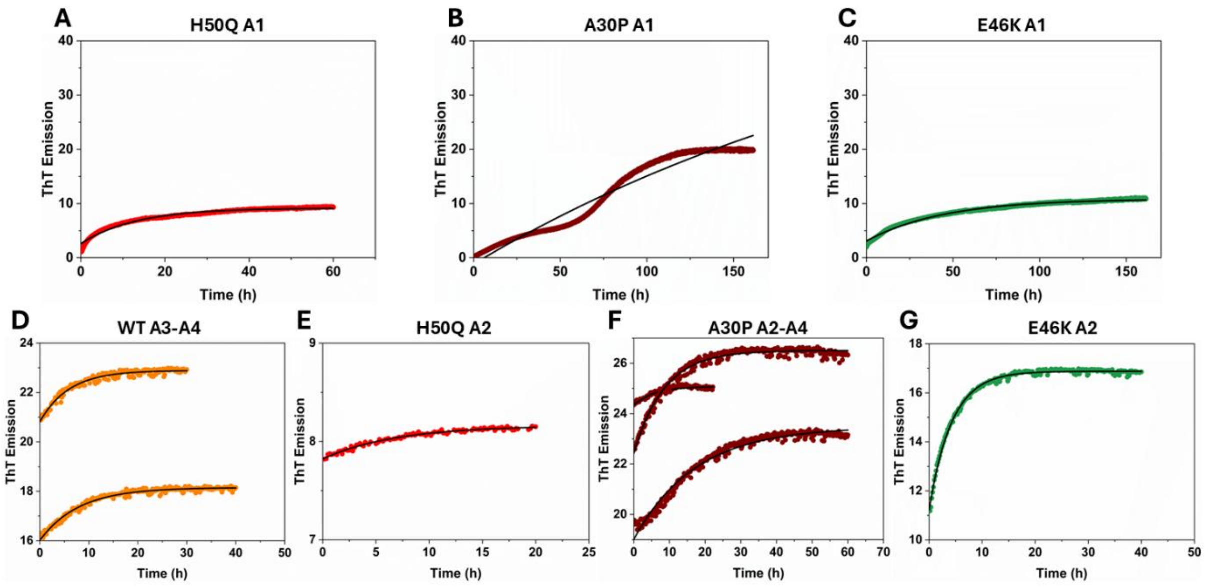

H

	y0	A	t_1	R^2	$[M]_{End}$	k_+ $[M^{-1}s^{-1}]$	$[M]_{Start}$	$\upsilon_{elongation}$ (nm/min)
WT A1	28732	-26086	10,45	0,999	4,54E-05	2087	5,00E-05	2,943
WT A3	22896	-2078	5,94	0.966	4,38E-05	1039	8,15E-06	0,239
WT A4	18150	-2135	7,96	0,979	4,23E-05	733	8,69E-06	0,180
H50Q A1	9191	-6584	13,16	0,975	4,55E-05	1155	5,00E-05	1,629
H50Q A2	8161	-339	6,80	0,980	4,70E-05	1342	6,99E-06	0,264
A30P A1	53836	-54988	286	0,955	3,19E-05	87	5,00E-05	0,123
A30P A2	25088	-739	5,87	0,919	3,62E-05	1899	2,06E-05	1,105
A30P A3	26507	-4090	8,53	0,981	3,45E-05	860	1,63E-05	0,396
A30P A4	23478	-4471	17,10	0,979	3,14E-05	442	1,8E-05	0,224
E46K A1	10870	-7780	44,77	0,990	1,10E-05	1649	5,00E-05	2,325
E46K A2	16882	-5671	4,26	0,995	3,21E-05	2367	4,16E-05	2,774
A53T A1	139239	-74987	27,78	0,959	3,29E-05	450	5,00E-05	0,635
A53T A2	185814	-58333	13,43	0,985	3,82E-05	742	1,96E-05	0,411
A53T A3	225649	-55533	18,17	0,994	3,61E-05	462	1,43E-05	0,187
A53T A4	236135	-36827	12,64	0,990	3,42E-05	622	1,64E-05	0,287

Figure S18. Fitting of ThT aggregation-kinetcs data, rate constants and elongation (nm/min). (A-C) H50Q, A30P and E46K seeded from monomer fluorescence spectra fitted to a single exponential function (black line). (D-G) WT, H50Q, A30P and E46K re-aggregation fluorescence spectra fitted to a single exponential function (black line). (H) Table with variables and constants for calculating the elongation rate (k+) and elongation per minute ($\upsilon_{elongation}$). y0 is the offset, A is the amplitude, t1 is the time constant, R2 is fit to data, $[M]_{End}$ is the concentration of fibrils at the end-point of aggregation and [M]Start is the concentration of monomers at the start of aggregation.

2. Supporting Information Text

Weibull distribution

The Weibull distribution, which accounts for both fragmentation and growth processes, has been shown to explain fibril length distributions in amyloid samples under fragmentation conditions. The Weibull probability density function is described by Eq. 1:

$$f(L) = \frac{k}{\lambda}\left(\frac{L}{\lambda}\right)^{k-1} * e^{-\left(\frac{L}{\lambda}\right)^{k}} \qquad (1)$$

Where L is the fibril length, λ is a scaling parameter (providing characteristic length of the system) and k is a shape parameter. If k<1, the probability of fragmentation decreases with increasing fibril length. If k = 1, the system is dominated by pure random, length-independent polymer breakage and the fibril length distribution becomes purely exponential. If k>1, fibril fragmentation probability increases with increasing fibril length, which is common in amyloid fibril systems, where longer fibrils are generally more prone to fragmentation than shorter ones.

Calculation of elongation rate constants.

A linear fit was initially attempted, but because the starting concentration of monomers and fibrils in aggregation phases A2-A4 cannot be accurately determined, and thus the data cannot be normalized to extract numerical elongation rates, we instead applied an exponential fit as described below. In this approach, the elongation rate is captured by the curve shape, while any offset is dealt with by the preexponential factor, avoiding the limitations on the linear fit.

To calculate the elongation rate constant k_+ [$M^{-1}s^{-1}$] and the growth per minute (nm/min) the ThT emission curves in Fig. 2 which showed increasing ThT intensity were fitted to a single exponential function (Eq. 2)

$$y = A * e^{\left(-\frac{x}{t1}\right)} + y0 \qquad (2)$$

Where A is the amplitude, 1/t1 [1/s] is the effective elongation constant ($k_{+,eff}$) and y0 is the offset. The concentrations and length of fibrils were measured at the end-point of the aggregation curves. A distance of 4.7 Å (0.47 nm) spacing between beta-sheets in the mature amyloid fibril and an architecture of two protofilaments were assumed to calculate the number of monomers/nm of fibril. The rate constant k_+ [$M^{-1}s^{-1}$] could then be calculated by dividing the effective elongation constant ($k_{+,eff}$) by the average number of monomers in each fibril.

Considering that amyloid fibrils grow unidirectional; the fibril growth per minute (nm/min) could be calculated by Eq. 3:

$$\upsilon_{\text{elongation}}\left(\frac{nm}{min}\right) = k_+ * [M]_{Start} * 4.7\text{Å} \qquad (3)$$

Where k_+ ($M^{-1}s^{-1}$) is the elongation rate constant, $[M]_{Start}$ is the monomer concentration at the start of each aggregation and re-aggregation, and 4.7 Å is the spacing between beta-sheet strands in the mature amyloid fibril.
